# Neuromuscular electrical stimulation in the intensive care unit prevents muscle atrophy in critically ill older patients: A retrospective cohort study

**DOI:** 10.1097/MD.0000000000029451

**Published:** 2022-08-05

**Authors:** Tadayoshi Nonoyama, Hiroko Shigemi, Masafumi Kubota, Akihiko Matsumine, Kenji Shigemi, Tamotsu Ishizuka

**Affiliations:** a Third Department of Internal Medicine, Faculty of Medical Sciences, University of Fukui, Eiheiji, Fukui, Japan; b Department of Rehabilitation, University of Fukui Hospital, Eiheiji, Fukui, Japan; c Division of Infection Control and Prevention, Faculty of Medical Sciences, Kyoto Prefectural University of Medicine, Kyoto, Japan; d Department of Physical Therapy, Graduate Course of Rehabilitation Science, School of Health Sciences, College of Medical, Pharmaceutical, and Health Sciences, Kanazawa University, Kanazawa, Japan; e Department of Orthopedics and Rehabilitation Medicine, Faculty of Medical Sciences, University of Fukui, Eiheiji, Fukui, Japan; f Department of Anesthesiology, Faculty of Medical Sciences, University of Fukui, Eiheiji, Fukui, Japan.

**Keywords:** intensive care unit, lower limb muscle atrophy, muscle thickness, neuromuscular electrical stimulation, older patients, physical function

## Abstract

Critically ill patients in the intensive care unit (ICU) develop muscle atrophy and decreased physical function. Though neuromuscular electrical stimulation (NMES) therapy has been shown to be effective in preventing this, but its effect on older patients is unknown.

To examine the course of critically ill older patients treated with NMES in the ICU and to define the impact of its use.

A retrospective cohort study was conducted using older ICU patients (≥65 years) categorized into a control group (n = 20) and an NMES group (n = 22). For subgroup analysis, each group was further classified into pre-old age (65–74 years) and old age (≥75 years).

The control group showed significant decrease in muscle thickness during ICU and hospital stay. The NMES group showed lower reduction in muscle thickness and showed decrease in muscle echo intensity during hospital stay, compared to the control group. NMES inhibited decrease in muscle thickness in the pre-old age group versus the old age group. The decreasing effect of NMES on echo intensity during hospital stay manifested only in the pre-old age group. We did not find much difference in physical functioning between the NMES and control groups.

Lower limb muscle atrophy reduces in critically ill older patients (≥65 years) with NMES and is pronounced in patients aged < 75 years. The impact of NMES on the physical functioning of older patients in ICU needs to be further investigated.

## 1. Introduction

Critically ill patients admitted in an intensive care unit (ICU) suffer from severe muscle weakness and atrophy referred to as ICU-acquired weakness (ICU-AW). The incidence of ICU-AW is reportedly 46% among critically ill patients,^[1]^ and decreased muscle thickness is associated with the onset of ICU-AW and physical functioning at discharge.^[2]^ Most critically ill patients have new onset of frailty, which is independently associated with mortality and impairment of activities of daily living (ADL).^[3]^ In addition, motor dysfunction persists for months to years^[4,5]^ and continues in 32% of patients.^[6]^ Preventing muscle atrophy and ICU-AW is essential for avoiding long-term motor dysfunction. In older patients, muscle atrophy, muscle weakness, and physical functioning decline progressively with age, even before ICU admission. The prevalence of sarcopenia increases with age,^[7]^ and skeletal muscle mass loss is associated with a greater decline in ADL.^[8]^ In particular, age is a risk factor for ICU-AW,^[9]^ and compared to younger patients, older patients are at a higher risk of muscle mass loss and ICU-AW due to pre-existing sarcopenia and anabolic resistance.^[10]^

Neuromuscular electrical stimulation (NMES) is used to treat skeletal muscle disorders in critically ill patients. Reportedly, NMES reduces muscle atrophy in critically ill patients,^[11–13]^ decreases the incidence of ICU-AW,^[14]^ improves muscle strength early, and reduces the duration of mechanical ventilation.^[15]^ The effects of NMES on older people have been reported, including an increase in cross-sectional areas of muscle tissue and muscle strength^[16]^ and improvements promoting postural control.^[17,18]^ However, there are no reports on its effects on older patients admitted to the ICU, and such effects are unclear. In addition to quantitative assessment, qualitative assessment of muscle mass is also attracting attention. Decreased muscle quality in critically ill patients is assessed by enhanced muscle echo intensity on ultrasound images.^[19,20]^ However, no studies have examined the effect of NMES on muscle quality, and the recovery rate of physical functioning is worse in older persons than in the young.^[5]^ Although frailty is a major concern in older adults following ICU discharge, the preventive effect of NMES has not been reported.

We hypothesized that NMES in critically ill older ICU patients effectively reduces lower limb muscle atrophy, promotes muscle quality and muscle strength, and improves physical functioning at the time of hospital discharge. Therefore, as a preliminary study, we retrospectively examined the course of critically ill older patients treated with NMES in the ICU to define the effects of NMES on muscle thickness (muscle volume), echo intensity of muscle (muscle quality), muscle strength, and physical function and evaluated its impact on older patients multilaterally.

## 2. Material and Methods

### 2.1. Study design

This is a single-center retrospective cohort study, and data were extracted from medical records and databases. The subjects were critically ill older ICU patients (≥65 years) in a Japanese tertiary hospital between June 1, 2018, and August 31, 2019. Echo measurements and physical function assessments were started with all patients on June 1, 2018, and NMES was introduced in the ICU on June 1, 2018. Therefore, patients admitted to the ICU between June 1, 2018, and January 31, 2019, before the introduction of NMES, formed the control group. Patients admitted to the ICU between February 1, 2019, and August 31, 2019, after NMES was introduced, were included in the NMES group.

The exclusion criteria were as follows: ICU stay <72 hours, mechanical ventilation <72 hours, not eligible for early mobilization and rehabilitation protocols, motor paralysis before hospitalization or ICU admission, case of bone fracture, case of death, patients with missing data, patients who failed to start NMES within 48 hours, those who had fewer than 3 NMES sessions, and those with swelling, wounds, or skin disorders at the measurement site of the ultrasound. As this was a retrospective study, patients with missing data were excluded.

### 2.2. Early mobilization

Early rehabilitation was initiated within 48 hours and performed for all the subjects by a team comprising physicians, nurses, and clinical engineering technicians, led by a physical therapist dedicated to the ICU. This therapy was performed according to the early mobilization and rehabilitation protocols of the hospital ICU (Supplemental Digital Content 1, http://links.lww.com/MD/G890) specifically to ensure standardized rehabilitation. A patient’s mobilization target was set at daily conferences. Under appropriate analgesia and sedation management, bedside exercises, walking practice during ventilatory control, and walking in the hospital using a wheelchair were conducted. After discharge from the ICU, rehabilitation was continued until discharge from the hospital.

### 2.3. Neuromuscular electrical stimulation

**Figure 1.**
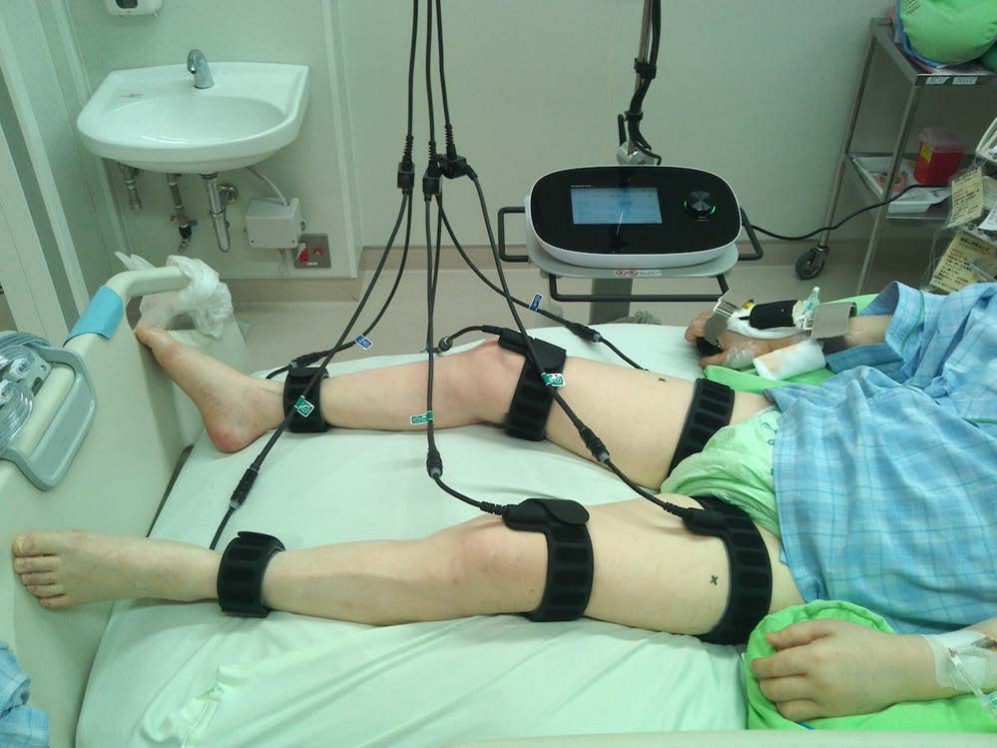
Neuromuscular electrical stimulation. Belt electrodes were attached to the proximal and distal thighs and ankles.

For NMES, belt electrode skeletal muscle electrical stimulation (G-TES; Homer Ion Corp., Osaka, Japan) was applied. Belt electrodes were attached to the patient’s proximal and distal thighs and ankles (Fig. 1). The NMES settings were set at a frequency of 20 Hz, a pulse width of 250 μs, and a duty cycle of 5 seconds of stimulation followed by 2 seconds of pause. The intensity of electrical stimulation was set at maximum intensity without pain, and muscle contraction was confirmed by inspection and palpation. NMES was started within 48 hours of ICU admission and applied once a day for 30 minutes, 5 days/wk, and only during ICU stay. The inclusion criteria for NMES were patients who were expected to stay in the ICU for >48 hours. The various contraindications were as follows: wearing a pacemaker, use of intra-aortic balloon pumping or support such as percutaneous cardiopulmonary support, skin damage at the electrode site, administration of muscle relaxants, and presence of deep vein thrombosis.

## 3. Outcome Measures

The primary outcome was muscle thickness, and secondary outcomes were echo intensity of muscle, grip strength, Medical Research Council (MRC) sum score, functional status score for the ICU (FSS-ICU), and short physical performance battery (SPPB).

### 3.1. Basic characteristics

The data collected as basic characteristics included age, gender, height, weight (at the time of ICU admission), clinical frailty scale (CFS) before admission, sequential organ failure assessment (SOFA) score (value on ICU admission, peak value), C-reactive protein (CRP) peak (value on ICU admission, peak value), emergency or plans, main diagnosis, number of days in the ICU and hospital, and days of mechanical ventilation.

### 3.2. Muscle thickness and muscle quality

**Figure 2.**
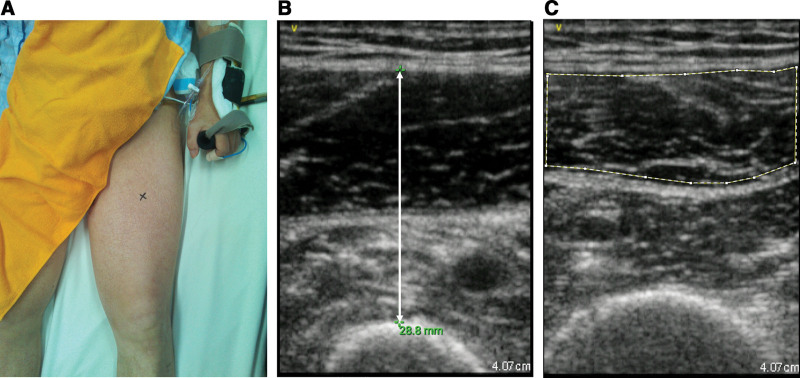
Measurement by ultrasound imagery. (A) Measurement site; (B) measurement of muscle thickness; (C) measurement area of echo intensity.

An ultrasound imaging system (Vscan Extend, GE Healthcare) was used to measure muscle thickness and echo intensity. The measurement positions were supine, mid-hip, and knee extension. The measurement site was the midpoint of the line connecting the superior anterior iliac spine and the upper patella (Fig. 2A). A 7.5-MHz linear probe was used to record the transverse ultrasound layer images when the deep probe was in perpendicular contact with the skin surface. Excess gel was used to minimize image distortion due to skin indentation caused by the pressure of the ultrasound probe.^[19,20]^ Muscle thickness was measured from the subcutaneous fat just above the femur (Fig. 2B), and echo intensity was used to evaluate muscle quality. Ultrasound transverse layer images of the rectus femoris muscle were measured with the Image J software (ver 1.53, National Institutes of Health, the United States) using the same images as those used for measuring muscle thickness. The measurement area was the region of interest (excluding fascia) of the rectus femoris muscle (Fig. 2C), which was evaluated using an 8-bit gray-scale in the range of 0 to 255, and the average value was used. In this connection, the echoes are attenuated in the deeper layers of tissue.^[21]^ It is difficult to use the echo intensity of deep muscle as a qualitative evaluation. Therefore, the echo intensity of the vastus medialis muscle was not measured in this study.

For subgroup analysis, to investigate the effect of age on muscle atrophy and muscle quality, the control and NMES groups were further classified into 2 groups, consistent with the definition proposed by the Joint Committee of Japan Gerontological Society and the Japan Geriatrics Society on the definition and classification of older people^[22]^: a pre-old age group (65–74 years old) and an old age group (≥75 years). In each group, muscle thickness and echo intensity were measured at the time of admission to the ICU, discharge from the ICU, and discharge from the hospital, and the changes were examined. In addition, the reduction rate in muscle thickness during ICU stay (from ICU admission to ICU discharge) and hospital stay (from ICU admission to hospital discharge) were calculated and compared between the control and NMES groups. The formula for the reduction rate of muscle thickness was as follows: reduction rate of muscle thickness during ICU stay = (ICU discharge − ICU admission)/ICU admission × 100. The ultrasound muscle measurements were performed by a dedicated ICU physiotherapist who was technically trained in the use of ultrasound equipment and had >2 years of experience performing measurements.

### 3.3. Muscle strength, basic motor function, and physical performance

At the time of discharge from the ICU and hospital, MRC sum score, grip strength (measured twice; and maximum value was used), and FSS-ICU were assessed for muscle strength and basic movement ability, respectively. SPPB was used to evaluate physical performance at the time of discharge from the hospital. The ICU-AW was defined as an MRC sum score of < 48 points, frailty was defined as an SPPB score of <9 points,^[23]^ and the onset of ICU-AW and frailty was evaluated.

### 3.4. Sample size

The study sample size was calculated by the method described by Gerovasili et al,^[11]^ who reported that the rate of change in rectus femoris muscle thickness was −8.0 ± 3.9% in the NEMS group and −13.9 ± 6.4% in the control group. GPower software (version 3.1) was used to determine the minimum sample size required for the study. An alpha level of 5% with a statistical power of 90% was included in the power analysis. Thus, a minimum of 18 patients were required per group.

### 3.5. Statistical analysis

Among the data collected, the Shapiro–Wilk test was used to check continuous variables for normality. Normally distributed indicators were expressed as mean ± standard deviation, and indicators that did not show normal distribution were expressed as median (interquartile range). Category variables were expressed as frequencies (%). The 95% confidence interval (95% CI) for the percentage reduction rate of muscle thickness was also shown. A one-way analysis of variance (ANOVA) and the Tukey post hoc test were performed to compare the changes in muscle thickness and echo intensity in each group at the time of ICU admission and discharge from the ICU and hospital. The unpaired t-test or Mann–Whitney U test compared the reduction rate of muscle thickness and echo intensity, muscle strength, and physical function between the 2 groups. A chi-square test was used for the qualitative variables of patient characteristics. The IBM SPSS software (version 27.0) was used for performing all measurements and calculations. A *P*-value <.05 was considered statistically significant.

### 3.6. Data analysis

The Research Ethics Committee of University of Fukui approved the study protocol (Assurance No. 20190136).

## 4. Results

### 4.1. Patient characteristics

**Figure 3.**
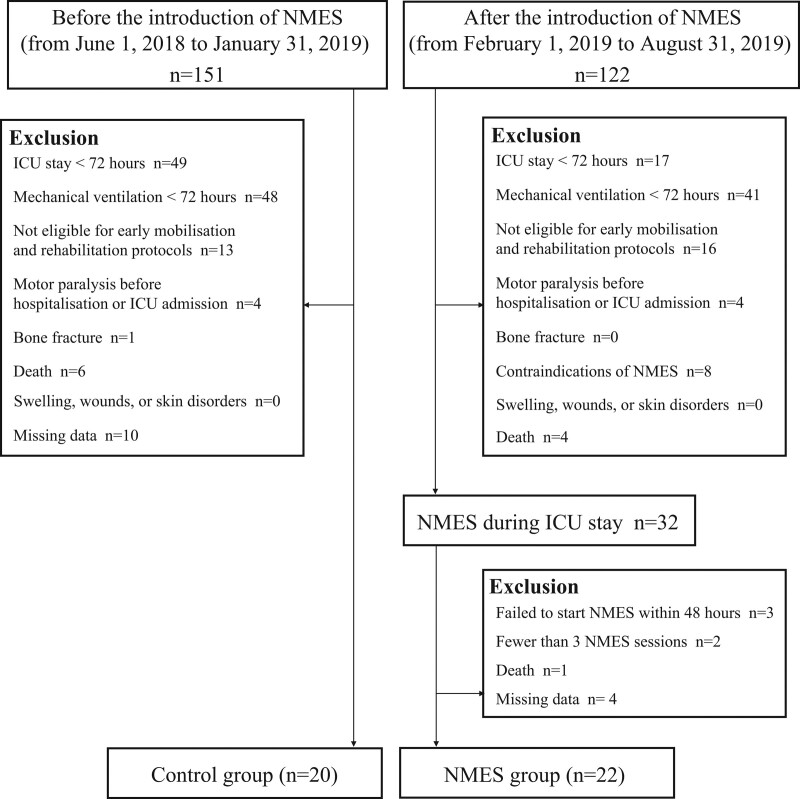
Flow diagram of the study. ICU = intensive care unit, NMES = neuromuscular electrical stimulation.

A total of 273 subjects were considered for analysis during the study period; 231 patients met the exclusion criteria, leaving 42 patients for analysis (Fig. 3). Table 1 presents the clinical and demographic characteristics of the patients. Twenty patients in the control group (78.0 [72.8 − 81.5] years, 13 men and 7 women) and 22 patients in the NMES group (78.0 [72.0 − 84.0] years, 22 men and 7 women) were included in the analysis. The number of patients at each stage of the early mobilization protocol at 48 hours after admission to the ICU was as follows: in the control group, step 1 included 11 patients, step 3 included 2 patients, and step 4 included 7 patients; in the NMES group, step 1 included 1 patient, step 2 included 3 patients, step 3 included 3 patients, step 4 included 3 patients, and step 5 included 2 patients. The subjects were followed up until they were discharged from the hospital. The follow-up period was 39.4 ± 17.7 days for the control group and 41.6 ± 20.5 days for the NMES group. Age, gender, height, weight (at the time of ICU admission), CFS before admission, SOFA score at ICU admission, peak SOFA score, peak CRP, admission type, surgical/internal medicine, number of days in the ICU, number of days in the hospital, and number of patients in each group were evaluated. There were no significant differences in the number of days in the ICU, number of days in the hospital, or number of ventilator days. The control group comprised 7 patients in the pre-old age group and 13 patients in the old age group. The NMES group had 10 patients in the pre-old age group and 12 patients in the old age group. Data on clinical and demographic characteristics showed no significant differences for the pre-old age group in the control and NMES groups and the old age group in the control and NMES groups (Supplemental Digital Content 2, http://links.lww.com/MD/G890).

**Table 1:** Clinical and demographic characteristics of patients

	Control group, n = 20	NMES group, n = 22	*P* value
Age, y, median [IQR]	78.0 [72.8 − 81.5]	78.0 [72.0 − 84.0]	.71
Male, n (%)	13 (65.0)	15 (68.2)	.82
Height, cm, mean ± SD	157.5 ± 10.2	158.6 ± 11.1	.73
Weight, kg, mean ± SD	56.4 ± 12.5	55.9 ± 13.2	.90
CFS, point	2.7 ± 1.1	3.1 ± 3.1	.20
SOFA, mean ± SD			
ICU admission	9.0 ± 2.9	9.1 ± 3.6	.90
Peak	10.1 ± 2.6	10.2 ± 3.2	.93
CRP peak, mL/dL, mean ± SD	20.4 ± 7.7	19.4 ± 10.2	.72
Type of ICU admission			
Emergency, n (%)	14 (70.0)	16 (72.7)	.84
Main diagnosis, n (%)			
Sepsis	6 (30.0)	10 (45.5)	.30
Abdominal/pelvic surgery	8 (40.0)	7 (31.8)	.58
Cardiac surgery	5 (25.0)	3 (13.6)	.35
Thoracic surgery	3 (15.0)	5 (22.7)	.52
Respiratory failure	1 (5.0)	3 (13.6)	.34
Heart failure	0	1 (4.5)	.33
Others	0	1 (4.5)	.33
ICU stay, d, mean ± SD	12.0 ± 5.5	12.6 ± 5.9	.70
Hospital stay, d, median [IQR]	33.5 [28.5 − 41.0]	38.0 [26.3 − 49.0]	.71
Mechanical ventilation, d, median [IQR]	8.5 [4.0 − 10.0]	8.5 [5.3 − 15.8]	.10

### 4.2. Muscle thickness

#### 4.2.1. Comparison of muscle thickness.

Muscle thickness at ICU admission, discharge, and hospital discharge showed no significant difference between the control and NMES groups (control group: admission 24.5 ± 6.1 mm; discharge from ICU 19.5 ± 6.1 mm; discharge from hospital 17.8 ± 5.7 mm, NMES group: admission 21.3 ± 6.0 mm; discharge from ICU 19.5 ± 5.9 mm; discharge from hospital 18.0 ± 6.1 mm).

In the pre-old age group, muscle thickness at ICU admission was significantly lower in the NMES group compared to the control group (control group 29.4 ± 4.1 mm vs NMES group 20.4 ± 5.9 mm, *P* < .05; Supplemental Digital Content 3, http://links.lww.com/MD/G890).

#### 4.2.2. Change in muscle thickness.

A significant decrease was observed in muscle thickness at the time of discharge from the ICU and hospital compared to ICU admission.

**Figure 4.**
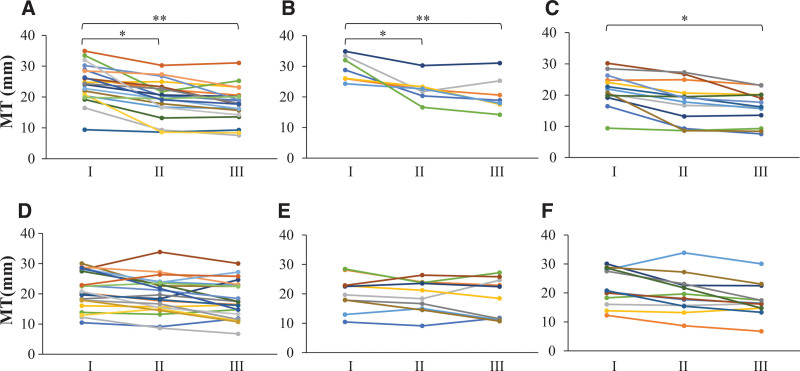
Change in muscle thickness. A significant decrease in muscle thickness was observed in the control group at the time of ICU and hospital discharge, compared to ICU admission (A). In the pre-old age group (B), a significant decrease in muscle thickness was observed at hospital discharge compared to ICU admission (C). The NMES group suppressed the decrease in muscle thickness (D–F). (A) All subjects in the control group; (B) pre-old age group of the control group; (C) old age group of the control group; (D) all subjects of the NMES group; (E) pre-old age group of the NMES group; (F) old age group of the NMES group; I. ICU admission; II. ICU discharge; III. Hospital discharge. *P < .05, **P < .01. ICU = intensive care unit, MT = muscle thickness, NMES = neuromuscular electrical stimulation.

1. Control group: admission 24.5 ± 6.1 mm versus discharge from ICU 19.5 ± 6.1 mm; *P* < .05 versus discharge from hospital 17.8 ± 5.7 mm, *P* < .01 (Fig. 4A).
2. Pre-old age control group: admission 29.4 ± 4.1 mm versus discharge from ICU 22.5 ± 4.0 mm; *P* < .05 versus discharge from hospital 20.7 ± 5.6 mm, *P* < .01 (Fig. 4B).
3. Old age control group: admission 21.9 ± 5.4 mm, discharge from hospital 16.2 ± 5.2 mm, *P* < .05 (Fig. 4C).

As determined by the one-way ANOVA, no significant differences were observed between the mean values of muscle thickness at the 3 assessment time points in the NMES group (Fig. 4D) or when the subjects were divided into pre-old and old age groups (Fig. 4E, F).

#### 4.2.3. Reduction rate in muscle thickness.

**Figure 5.**
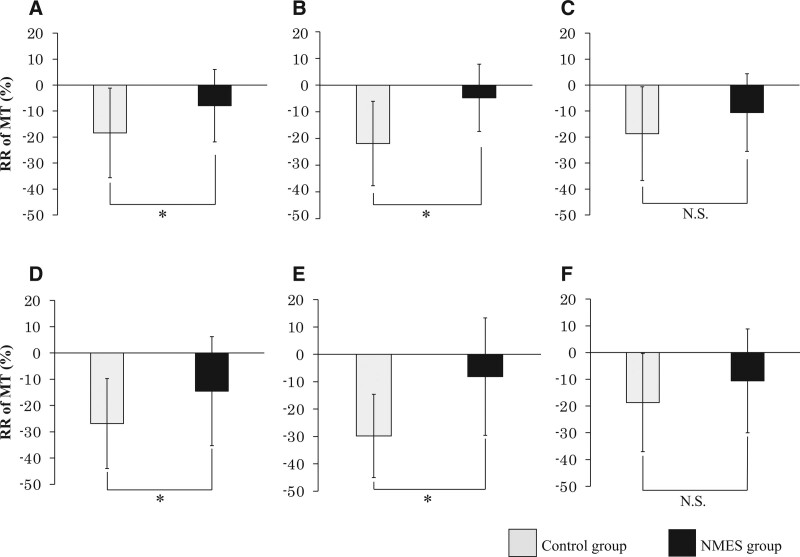
Reduction rate in muscle thickness. Application of NMES inhibited decrease in muscle thickness during ICU stay (A) and hospital stay (D). This inhibition was observed in the pre-old age group (B, E) but not the old age group (C, F). (A) All subjects during ICU stay; (B) pre-old age group during ICU stay; (C) old age group during ICU stay; (D) all subjects during hospital stay; (E) pre-old age group during hospital stay; (F) old age group during hospital stay. *P < .05, MT = muscle thickness, NMES = neuromuscular electrical stimulation, N.S.= not significant, RR = reduction rate.

The reduction rate in muscle thickness during ICU and hospital stay was −19.8% (95% CI −27.7 to −11.8) and −26.8% (95% CI −34.6 to −19.0) in the control group; −7.9% (95% CI −14.1 to −1.0) and −14.5% (95% CI −23.7 to −5.3) in the NMES group, respectively. The decline in muscle thickness during ICU stay was significantly lower in the NMES group, compared to the control group. The reduction rate in muscle thickness during hospital stay was suppressed drastically in the NMES group (*P* < .05; Fig. 5A, D). The pre-old age group showed a considerably suppressed reduction rate in muscle thickness in the NMES group during ICU stay (−21.8% [95% CI −36.5 to −7.2] vs −4.7% [95% CI −13.8 to −4.3], *P* < .05) and hospital stay (−29.3% [95% CI −42.2 to −16.4] vs −8.1% [95% CI −23.5 to −7.2], *P* < .05; Fig. 5B, E). The significantly suppressed reduction rate in muscle thickness observed in the NMES group was not identified in the old age group (Fig. 5C, F).

### 4.3. Echo intensity of muscle

**Figure 6.**
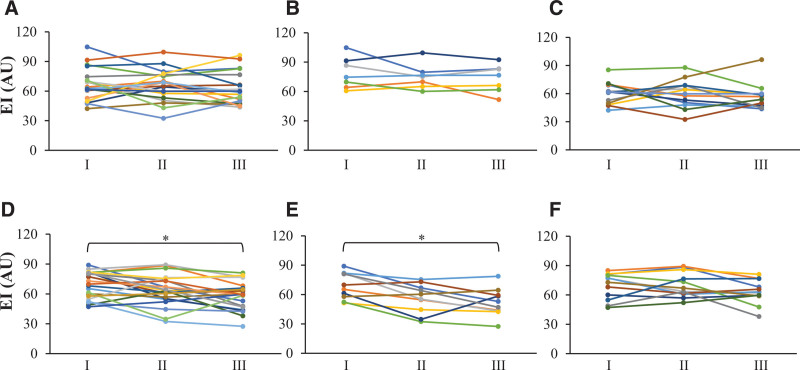
Change in echo intensity of muscle. Muscle echo intensity did not change in the control group (A, B, C) during the hospital stay. It decreased during the hospital stay in the NMES group (D). A significant decrease was observed in the pre-old age group (E) but not the old age group (F). (A) All subjects in the control group; (B) pre-old age group of the control group; (C) old age group in control group; (D) all subjects of the NMES group; (E) pre-old age group of the NMES group; (F) old age group of the NMES group; I. ICU admission; ICU discharge; Hospital discharge. *P < .05. EI = echo intensity, NMES = neuromuscular electrical stimulation.

In the control group, no major change in echo intensity was observed (Fig. 6A–C). In the NMES group, the echo intensity of muscle decreased significantly at the time of hospital discharge compared to ICU admission (admission 69.3 ± 13.2 AU vs discharge from hospital 58.0 ± 14.1 AU, *P* < .05; Fig. 6D). Notably, NMES reduced the echo intensity of muscle drastically in the pre-old age group; however, the same was not observed in the old age group (admission 69.2 ± 13.6 AU vs discharge from hospital 51.8 ± 14.2 AU, *P* < .05; Fig. 6E, F). The echo intensity of muscle at ICU admission, discharge, and hospital discharge showed no significant difference between the control and NMES groups (control group: admission 66.7 ± 16.0 AU; discharge from ICU 65.1 ± 15.7 AU; discharge from hospital 62.8 ± 15.7 AU, NMES group: admission 69.3 ± 13.2 AU; discharge from ICU 63.6 ± 15.1 AU; discharge from hospital 58.0 ± 14.1 AU). In the pre-old age group, echo intensity of muscle at ICU discharge and hospital discharge was significantly lower in the NMES group compared to the control group (Control group 73.5 ± 14.2 AU vs NMES group 51.8 ± 14.2 AU, *P* < .05; Supplemental Digital Content 3, http://links.lww.com/MD/G890).

### 4.4. Physical function and onset of ICU-AW and frailty

Table 2 presents details of physical functioning and onset of ICU-AW and frailty. There were no significant differences in the FSS-ICU, grip strength, or MRC sum score at the time of ICU discharge or hospital discharge or in SPPB score at hospital discharge. In addition, there was no significant difference observed in the onset of ICU-AW and frailty.

**Table 2:** Muscle strength, basic motor function, physical performance, and onset of ICU-AW and frailty.

	Control group, n = 20	NMES group, n = 22	*P* value
MRC sum score, median [IQR]			
Discharge from ICU			
Upper extremity	25.0 [22.8−28.0]	24.0 [22.2−27.5]	.54
Lower extremity	23.5 [21.8−26.5]	26.0 [21.3−26.8]	.89
Total score	49.0 [44.8−56.0]	47.6 [42.0−54.0]	.53
Discharge from Hospital			
Upper extremity	27.0 [24.0−28.5]	28.0 [24.0−30.0]	.40
Lower extremity	26.0 [23.8−28.0]	27.5 [24.5−28.0]	.63
Total score	52.0 [48.0−56.3]	53.2 [49.3−58.0]	.49
Grip strength, kg, mean ± SD			
Discharge from ICU	17.8 ± 6.4	16.2 ± 7.3	.45
Discharge from Hospital	18.7 ± 6.1	18.5 ± 7.0	.93
FSS − ICU, median [IQR]			
Discharge from ICU			
Rolling	3.5 [3.0−6.0]	3.0 [2.0−5.0]	.35
Supine to sit transfer	4.0 [3.0−4.5]	3.0 [2.3−4.8]	.33
Sitting on the edge of bed	5.5 [5.0−7.0]	5.0 [5.0−7.0]	.53
Sit to stand transfer	6.0 [4.0−6.0]	4.5 [3.0−6.0]	.33
Walking	5.0 [3.0−6.0]	4.5 [3.0−5.0]	.41
Total score	22.9 [19.3−27.3]	21.0 [13.3−27.5]	.45
Discharge from Hospital			
Rolling	7.0 [6.0−7.0]	7.0 [6.0−7.0]	.55
Supine to sit transfer	7.0 [6.0−7.0]	6.0 [6.0−7.0]	.23
Sitting on the edge of bed	7.0 [7.0−7.0]	7.0 [7.0−7.0]	.12
Sit to stand transfer	7.0 [6.0−7.0]	6.0 [6.0−7.0]	.22
Walking	6.5 [6.0−7.0]	6.0 [6.0−7.0]	.65
Total score	24.0 [19.3−27.3]	20.5 [13.3−27.5]	.42
SPPB, median [IQR]			
Balance	4.0 [1.8−4.0]	3.0 [2.0−4.0]	.95
Gait	3.5 [3.0−4.0]	3.0 [3.0−4.0]	.83
Stand up	3.0 [0−3.3]	1.0 [0−3]	.42
Total score	9.5 [5.0−11.0]	7.5 [6.0−9.8]	.57
ICU-AW, n (%)			
Discharge from ICU	9 (45.0)	11 (50.0)	.77
Discharge from hospital	4 (20.0)	3 (13.6)	.69
Frailty, n (%)	10 (50.0)	13 (59.1)	.55

## 5. Discussion

Our study shows that NMES inhibited reduction in muscle thickness during ICU stay and prevented muscle atrophy in critically ill older patients. With the aging population and advances in medical technology, the number of older patients receiving intensive care is increasing. Muscle atrophy in the ICU may accelerate the onset and progression of sarcopenia and frailty, leading to an increased burden of care and medical costs. Thus, this is a social problem and an urgent issue. The fact that NMES effectively prevents muscle atrophy in older ICU patients is of significance because it will help address these problems. As a mechanism by which NMES was effective in inhibiting muscle atrophy, the studies on the effects of NMES with a large number of older subjects reported a decrease in branched-chain amino acids^[13]^ and a decrease in urinary excretion of 3-methylhistidine,^[24]^ an indicator of protein catabolism; the same was expected to be true when only older subjects are included in the study. However, when subjects were categorized into the pre-old and old age groups, the effect was observed only in the pre-old age group and not in the old age group. It is speculated that age-related decreases in growth hormone and insulin-like growth factor-1 concentrations^[25]^ may influence NMES-related inhibition of muscle atrophy in older patients; however, the factors are unclear, as differences in muscle protein catabolism and responsiveness to NMES in different age groups of older patients are not known. This mechanism needs to be clarified in the future.

There were no significant differences in muscle strength and physical functioning at all of the endpoints between the NMES and control groups. Although some reports include many older people in the target,^[12]^ there are no detailed evaluations of the impact of EMS on physical functioning. In previous reports that showed significant improvement in muscle strength by NMES, no active rehabilitation was performed in the control group.^[15,26,27]^ In contrast, early rehabilitation was performed in the control group in a study that did not demonstrate improvement in muscle strength after exercise intensification using NMES or in-bed ergometer.^[28–30]^ In this study, all subjects experienced active weaning and rehabilitation based on the early weaning protocol; at the time of ICU discharge, the FSS-ICU was approximately 21 to 22 points, and weaning using the lower limbs, such as standing and walking, had progressed. In our study, the effect of early mobilization may have masked the impact of NMES on muscle strength and physical functioning. There may be several reasons for NMES changing muscle thickness but having no effect on strength or performance. The MRC sum score assessed lower extremity muscle strength because it could not capture changes in muscle strength as it was not evaluated in detail. In addition, NMES suppressed atrophy of the quadriceps muscle; however, as only the latter was assessed by ultrasound imaging, it is possible that atrophy in other muscles was not inhibited. As a result, it may be deduced that NMES did not affect physical functioning. As muscle strength and physical functioning did not differ in the NMES group from those in the control group, the incidence of ICU-AW and frailty did not vary between the 2 groups. As one of the major goals of rehabilitation in the ICU is minimizing the incidence of ICU-AW and frailty, the exclusive addition of NMES may have little impact on reducing the incidence of these conditions. In ICU patients, muscle atrophy occurs early and rapidly within a few days of admission.^[2]^ Muscle mass loss during ICU stay is a factor in post-ICU functional impairment.^[19]^ Increased quadriceps thickness during hospitalization in ICU patients is associated with improved physical function 3 months after discharge.^[31]^ Controlling muscle atrophy from the time of ICU admission may be of value for long-term physical function. More studies on rehabilitation to reduce the incidence of ICU-AW and frailty are needed in the future.

In the NMES group, echo intensity of skeletal muscle decreased at the time of hospital discharge rather than at admission. An examination of the results by age group shows a significant decrease in the pre-old age group but not in the old age group, similar to the trend in muscle thickness. Echo intensity in skeletal muscle reflects intramuscular adipose tissue and interstitial fibrous tissue,^[32,33]^ and echo intensity increases in atrophied muscles.^[19]^ In addition, echo intensity diminishes with strength training,^[34]^ enabling the capture of qualitative changes in muscles. In this study, changes were observed only in the NMES group, reflecting the qualitative improvement in muscle due to the treatment. However, it is unlikely that intramuscular fat would be reduced at the time of hospital discharge, compared to at admission. It is hypothesized that muscle quality had already decreased at the time of first ultrasound imaging evaluation compared to before hospitalization. The subsequent improvement process may have been captured. Future studies can verity the effects on qualitative changes in muscles.

One limitation of this study is that the data were collected retrospectively and thus the methodological quality is not high enough. Although there were no significant differences in patient background between the 2 groups, the possibility of bias due to unmeasured factors cannot be ruled out because this was not a randomized controlled trial. The study is based on data collected at a single hospital, and the small number of subjects and especially the small sample size for the subgroup analysis may make it unreliable, although significant differences were found. The large number of septic and postoperative patients introduced disease bias. Therefore, external validity is limited; further case data collection and prospective multicenter studies are needed to confirm the efficacy of NMES in elderly critically ill patients admitted to the ICU. In addition, only 1 person was measured by ultrasound and was not blinded. Future prospective studies should be blinded by multiple evaluators to ensure higher quality. We used muscle thickness as an indicator of muscle atrophy, and it may be underestimated because measuring the cross-sectional muscle areas is superior to muscle thickness in assessing muscle atrophy.^[35]^ Although it was difficult to capture the entire muscle cross-section due to the equipment used, we captured the changes in muscle thickness.

## 6. Conclusion

Critically ill older patients who underwent neuromuscular electrical stimulation in the ICU showed less reduction in muscle thickness. It is proposed that NMES during ICU stay may reduce lower limb muscle atrophy in these patients. However, it did not seem to be effective in older patients (>75 years). In addition, the effect of NMES on physical function at discharge from the hospital is not clear and needs to be tested in future studies.

## Acknowledgments

The authors are grateful to the ICU doctors and nurses, namely Dr Michiko Kitamura, Dr Ritsuko Saito, Mr. Shingo Haneda, Mr. Takeshi Okamoto, Mr. Yuji Kuwabara, for extending their support and cooperation toward fulfillment of this study’s objectives. We appreciate Dr Takahiro Tokunaga of the Medical Research Support Center, University of Fukui Hospital for his advice on statistical analysis.

## Author contributions

Conceptualization: T.N., H.S., M.K., A.M., K.S., T.I.

Data curation: T.N., H.S.

Formal analysis: T.N., H.S., M.K.

Funding acquisition: T.N.

Investigation: T.N., H.S., T.I.

Project administration: T.I.

Supervision: T.I.

Visualization: T.N., H.S., M.K.

Writing–original draft: T.N.

Writing–review & editing: H.S., M.K., A.M., K.S., T.I.

The final manuscript has been read and all authors have agreed to the content of the manuscript and acknowledge that all those entitled to authorship are listed as authors.

## Supplementary Material


