# Supplementary material for: Neuromuscular electrical stimulation in the intensive care unit prevents muscle atrophy in critically ill older patients: A retrospective cohort study
Source: Medicine (Baltimore). 2022 Aug 5;101(31):e29451. doi: 10.1097/MD.0000000000029451 (PMC9351912; doi:10.1097/MD.0000000000029451)
Supplement: Supplementary file 1 [file medi-101-e29451-s001.pdf]

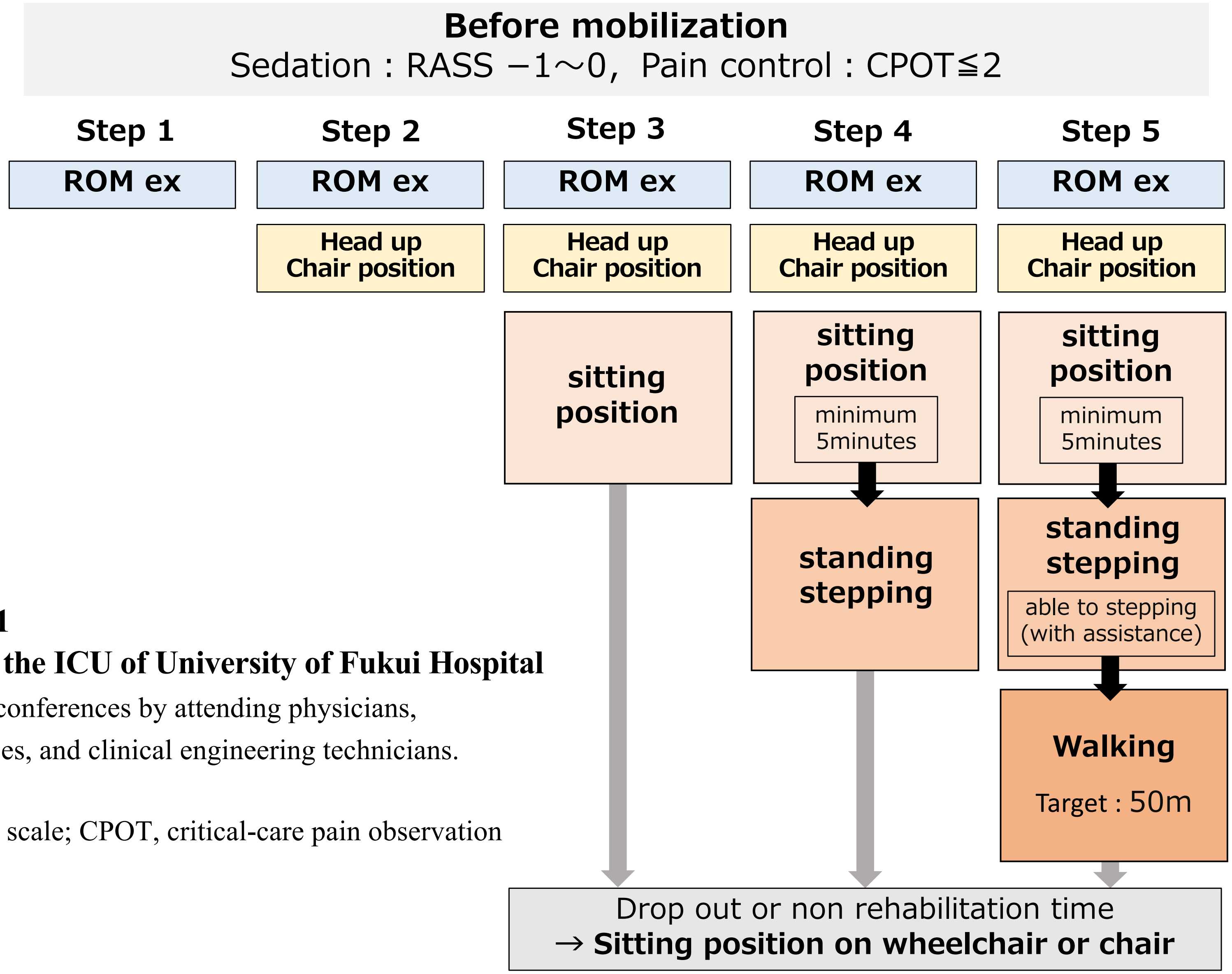

Supplemental Digital Content 1

Early Mobilization Protocol in the ICU of University of Fukui Hospital

Target mobilisation was set at daily conferences by attending physicians, intensivists, physical therapists, nurses, and clinical engineering technicians.

RASS, Richmond agitation-sedation scale; CPOT, critical-care pain observation tool; ROM, range of motion.

	Pre-old age			Old age		
	Control group n = 7	NMES group n = 10	P value	Control group n = 13	NMES group n = 12	*P* value
Age, years, mean ± SD	70.4 ± 3.0	70.8 ± 3.3	0.81	81.6 ± 4.6	83.8 ± 3.1	0.18
Male, n (%)	3 (42.8)	8 (80.0)	0.11	10 (76.9)	7 (58.3)	0.32
Height, cm, mean ± SD	158.6 ± 7.6	164.3 ± 8.2	0.97	156.8 ± 11.7	153.8 ± 11.3	0.52
Weight, kg, mean ± SD	61.8 ± 9.9	57.6 ± 13.4	0.49	53.5 ± 13.1	54.5 ± 13.5	0.86
Clinical Fraility Scale, point	2.71 ± 1.0	2.8 ± 1.2	0.87	2.6 ± 1.3	3.4 ± 1.5	0.17
SOFA, mean ± SD						
ICU admission	8.71 ± 2.4	9.3 ± 4.6	0.76	9.1 ± 3.2	9.0 ± 2.8	0.95
Peak	10.1 ± 2.5	10.8 ± 3.7	0.69	10.2 ± 2.5	9.8 ± 2.8	0.71
CRP peak, ml/dl, mean ± SD	21.2 ± 10.2	19.3 ± 11.4	0.73	20.0 ± 6.5	19.5 ± 9.6	0.88
Type of ICU admission						
Emergency, n (%)	5 (71.4)	6 (0.60)	0.62	9 (69.2)	10 (83.3)	0.41
Main diagnosis, n (%)						
Sepsis	0	1 (10.0)	0.39	3 (23.0)	1	0.32
Abdominal / pelvic surgery	4 (57.1)	1 (10.0)	0.12	2 (15.4)	6 (50.0)	0.06
Cardiac surgery	1 (14.3)	1 (10.0)	0.78	6 (46.2)	2 (16.6)	0.11
Thoracic surgery	1 (14.3)	4 (40.0)	0.25	2 (15.4)	1 (8.3)	0.59
Respiratory failure	1 (14.3)	1 (10.0)	0.78	0	2 (16.6)	0.12
Heart failure	0	1 (10.0)	0.39	0	0	
Others	0	1 (10.0)	0.39	0	0	
ICU stay, days, mean ± SD	13.6 ± 7.3	13.9 ± 6.2	0.92	11.1 ± 4.4	11.6 ± 5.7	0.81
Hospital stay, days, median [IQR]	40.7 ± 16.1	42.3 ± 18.6	0.86	38.8 ± 19.1	41.1 ± 22.7	0.78
Mechanical ventilation, days, median [IQR]	10.6 ± 5.7	11.9 ± 6.4	0.67	6.9 [5.0−9.0]	13 [5.0−14.5]	0.14

Supplemental Digital Content 2. Clinical and demographic characteristics data of pre-old and old age

Normally distributed indicators were expressed as mean ± standard deviation (SD), and indicators that did not show normal distribution were expressed as medians (interquartile range [IQR]).

CFS, clinical frailty scale; SOFA, sequential organ failure assessment; ICU, intensive care unit; CRP, C-reactive protein

	Pre-old age			Old age		
	Control group n = 7	NMES group n = 10	P value	Control group n = 13	NMES group n = 12	*P* value
Muscle thickness (mm), mean ± SD						
ICU admission	29.4 ± 4.1	20.4 ± 5.9	0.003	21.8 ± 5.4	22.0 ± 6.3	0.95
ICU discharge	22.5 ± 4.1	19.2 ± 5.4	0.2	17.8 ± 6.4	19.7 ± 6.7	0.48
Hospital discharge	20.8 ± 5.6	18.6 ± 6.8	0.5	16.2 ± 5.2	17.4 ± 5.8	0.58
Echo intensity of muscle (AU), mean ± SD						
ICU admission	78.8 ± 16.0	69.2 ± 13.6	0.2	60.1 ± 12.0	69.5 ± 13.5	0.08
ICU discharge	75.1 ± 12.7	56.1 ± 15.0	0.02	59.7 ± 14.8	69.9 ± 12.7	0.08
Hospital discharge	73.5 ± 14.2	51.8 ± 14.2	0.007	57.0 ± 13.5	63.1 ± 12.2	0.25

Supplemental Digital Content 3. Muscle thickness and echo intensity of muscle of pre-old and old age

NMES, neuromuscular electrical stimulation; ICU, intensive care unit
